# Virus-mediated, heritable gene editing in groundcherry (*Physalis grisea*)

**DOI:** 10.3389/fpls.2026.1794888

**Published:** 2026-03-20

**Authors:** Redeat Tibebu, Evan E. Ellison, Sydney R. Winecke, Lynn E. Prichard, Nick Klejeski, Lilee I. Donahue, Jon P. Cody, Can Baysal, Colby G. Starker, Joyce Van Eck, Daniel F. Voytas

**Affiliations:** 1 Department of Genetics, Cell Biology and Development, University of Minnesota, Saint Paul, MN, United States; 2 Center for Precision Plant Genomics, University of Minnesota, Saint Paul, MN, United States; 3 Department of Horticultural Sciences, University of Florida, Gainesville, FL, United States; 4 Boyce Thompson Institute for Plant Science, Ithaca, NY, United States; 5 Plant Breeding and Genetics Section, School of Integrative Plant Science, Cornell University, Ithaca, NY, United States

**Keywords:** CRISPR/Cas9, gene editing, groundcherry, polyploid, virus-induced gene editing

## Abstract

**Introduction:**

Virus-induced gene editing (VIGE) provides a powerful alternative to conventional plant genome engineering by enabling in planta delivery of genome-editing reagents without repeated use of tissue culture. Here, we establish Tobacco rattle virus (TRV)–mediated VIGE as an efficient system for somatic and heritable genome editing in groundcherry (*Physalis grisea*).

**Methods:**

Using Cas9-expressing plants, we targeted the visual marker gene *PHYTOENE DESATURASE* (PDS) and the domestication gene *CLAVATA1* (CLV1) via TRV-mediated delivery of guide RNAs. Editing efficiencies were evaluated in somatic tissues and across progeny to assess heritability.

**Results:**

Targeting of PDS resulted in somatic editing frequencies of 80–95% and consistent recovery of heritable edits, with all infected plants (n = 5) producing edited progeny, including fully albino seedlings carrying frameshift mutations in all alleles. The primary Cas9-expressing line was unexpectedly tetraploid, likely due to genome duplication during tissue culture. Despite this, VIGE efficiently generated mono-, bi-, tri-, and tetra-allelic mutations, demonstrating robust editing across four alleles simultaneously. Targeting of *CLV1* achieved somatic editing frequencies of up to 73%, with 60% of T0 plants producing heritable edits. Edited plants exhibited increased floral organ number and multilocular fruits, consistent with *CLV1* loss-of-function phenotypes.

**Conclusion:**

These results demonstrate that VIGE enables rapid, efficient, and heritable genome editing in groundcherry, even in a tetraploid context, highlighting its potential to accelerate genetic improvement and de novo domestication of underutilized crops.

## Introduction

The potential of gene editing to revolutionize agriculture and food systems is well recognized. Over the past decade, numerous studies have demonstrated the effectiveness of gene editing in both model plant systems and major crop species (Cardi et al., 2023; Chen et al., 2019; Jaganathan et al., 2018). However, the application of these technologies to a broader range of crops – particularly neglected and underutilized species (NUS) – has been limited, largely due to the absence of reference genomes and efficient plant transformation protocols (Shorinola et al., 2024; Swain et al., 2025; Venezia and Creasey Krainer, 2021). NUS play a crucial role in the Global South as primary or supplementary food sources and often possess unique nutritional profiles that address deficiencies common in major crops. In addition, their resilience to harsh environments and low-input agricultural systems makes them attractive candidates for climate-change adaptation and for supporting the economic advancement of subsistence farmers (Ndlovu et al., 2024; Shafiq et al., 2025; Tadele, 2019; Talabi et al., 2022; Verbeecke et al., 2023). Despite these advantages, many NUS suffer from low productivity due to traits such as indeterminate growth, small fruit size, and fragile stem or root structures that predispose plants to lodging (Akpojotor et al., 2025; Chacha et al., 2022; Huang et al., 2025; Tadele, 2019).

Groundcherry (*Physalis grisea*), the focus of this study, is a fruit crop in the Solanaceae family indigenous to North America (Dale et al., 2024). Although its semi-sweet berries have potential for large-scale commercial production, several undesirable traits – including small fruits enclosed in a papery husk, indeterminate growth habit, and extensive fruit drop – limit its appeal to growers and consumers (Dale et al., 2024; Van Eck, 2022). Recent advances in genome sequencing technologies have begun to alleviate barriers to the genetic improvement of NUS. High-quality, chromosome-scale reference genomes for *P. grisea* and *P. pruinosa* have recently been generated and made publicly available (He et al., 2023). In parallel, the establishment of a conventional *Agrobacterium*-mediated transformation protocol for groundcherry has enabled the application of CRISPR/Cas9-mediated gene editing to introduce domestication traits (Lemmon et al., 2018; Swartwood and Van Eck, 2019).

While initial studies underscore the promise of CRISPR/Cas9 for the *de novo* domestication of groundcherry, they rely on conventional transformation approaches that require tissue culture. Tissue culture remains an inefficient and labor-intensive process that is highly species dependent and can induce undesirable genetic and epigenetic alterations (Altpeter et al., 2016). An alternative strategy for delivering genome-editing reagents exploits plant viruses, which can systemically transport nucleic acids throughout infected plants (Steinberger and Voytas, 2025). Tobacco rattle virus (TRV) offers several advantages as a viral vector, including mild symptoms in most host species and the ability to infect meristematic tissues (Martín-Hernández and Baulcombe, 2008; Ratcliff et al., 2001).

In previous work, we demonstrated high frequencies of heritable genome editing in Cas9-expressing *Nicotiana benthamiana*, *Arabidopsis* and tomato plants using TRV vectors to deliver mobile single guide RNAs (Ellison et al., 2020; Liu et al., 2024; Nagalakshmi et al., 2022). In this study, we applied VIGE to achieve both somatic and heritable genome editing in Cas9-expressing groundcherry lines. We first validated this delivery system by targeting the *PHYTOENE DESATURASE* gene, whose disruption results in a photobleached phenotype (Qin et al., 2007). We then targeted the domestication gene *CLAVATA1*, which plays a central role in regulating the floral meristem and determining fruit size (Lemmon et al., 2018; Xu et al., 2015). Together, these results demonstrate a streamlined approach for genome editing that holds significant promise for the *de novo* domestication and genetic improvement of groundcherry and other NUS.

## Materials and methods

### Cas9-expressing groundcherry lines

Two different constructs, pTC232 and pNJB193, were transformed into *P. grisea* by *Agrobacterium*-mediated transformation (Swartwood and Van Eck, 2019). These constructs express Cas9 from the 35S promoter and have previously been used to create tomato and *Nicotiana benthamiana* lines for VIGE (Čermák et al., 2015; Ellison et al., 2020). DNA was extracted from 4mm leaf punches from each putative transgenic (Doyle and Doyle, 1990). The presence of Cas9 was assessed by PCR amplification of the genomic DNA samples using primers specific to Cas9 (**Supplementary Table 1**). PCR was performed with either the Q5 polymerase kit (New England Biolabs) or the PHIRE Plant Direct PCR Kit (Thermo Fisher). The same PCR conditions were used for both polymerases: initial denaturation at 95 °C for 1 min followed by 35 cycles of denaturation at 95 °C for 12 sec, annealing at 64-65 °C for 15 sec, and extension at 72 °C for 15 sec. The final extension was carried out at 72 °C for 5 min. Using methods previously described, immunoblot analysis was performed on groundcherry lines that were PCR-positive for Cas9 to assess Cas9 protein expression (Baysal et al., 2025). Flow cytometry to assess ploidy levels in the transformed lines was performed as described (Loureiro et al., 2023).

### sgRNA design and tobacco rattle virus vector assembly

sgRNAs targeting *P. grisea PDS* (sgRNA1 and sgRNA2) were designed using the *P. pruinosa PDS* gene as a reference (SRX39974000) (He et al., 2023) and the Cas-OFFinder tool from CRISPR RGEN tools (Bae et al., 2014). sgRNA1, targeting *CLV1*, was previously described (Lemmon et al., 2018), whereas sgRNA2 and sgRNA3 were newly designed for this study (**Supplementary Table 2**).

Coding sequence for the AmCyan protein from *Anemonia majano* (Henderson and Remington, 2005) was cloned behind the Pea early browning virus promoter in TRV2. To construct TRV2 vectors with single or multiple mobile sgRNAs, primers were designed to amplify the sgRNA scaffold and the truncated FT sequence from pEE515 (Ellison et al., 2020) (**Supplementary Table 3**). The forward primer included a 20 bp spacer sequence at the 5’ end. Both forward and reverse primers were designed with either BsaI or AarI restriction enzyme sites and overhangs compatible with Golden Gate (GG) cloning into the TRV2 T-DNA backbone (pEE083) (Ellison et al., 2020; Engler et al., 2008). The GG reaction was performed using AarI for all TRV2 vectors, except for pRT327, which was assembled using a mixed GG reaction of AarI and BsaI.

For vectors with two sgRNAs, the respective amplicons with compatible overhangs were added to the GG reaction at a 1:2 ratio with pEE083. All reaction mixtures were run with 10 cycles of digestion (37 °C) and ligation (16 °C) for 5 and 10 min, respectively. A final digestion step was performed for 10 min, followed by enzyme inactivation at 80 °C for 5 minutes. All resulting clones were verified by DNA sequencing. Correctly assembled TRV2 vectors were transformed into *Agrobacterium tumefaciens* strain GV3101 using the freeze-thaw method (Weigel and Glazebrook, 2006).

### Agroinfiltration of Cas9-expressing groundcherry lines

GV3101 cultures harboring vectors of interest were propagated in LB medium overnight. The cultures were centrifuged for 10 min at a relative centrifugal force of 2,500 x g for 10 min, after which the supernatant was discarded, and the pellet resuspended in 4 ml of agroinfiltration buffer (10 mM MgCl2, 10 mM 2-(N-morpholino) ethanesulfonic acid (MES), pH 5.6). The centrifugation and resuspension steps were repeated twice, and the final resuspension was adjusted to an OD_600_ of 0.6. TRV1 and TRV2 cultures were diluted 1:1 and incubated at room temperature for 1 hour. An airtight 1 ml needleless syringe was used to infiltrate the adaxial side of groundcherry seedling cotyledons with the TRV1:TRV2 mixtures. Seedlings were grown under a 12-hour light cycle (80 to 100 μmol/m2s-1), 70% relative humidity, a night temperature of 22 °C and day temperatures of either 22 °C or 26 °C.

### Confirmation of somatic and germline gene editing

Gene editing frequencies were assessed in systemically infected leaves fourteen days post infection (dpi). Leaf samples were collected in 2 mL microcentrifuge tubes and flash frozen in liquid N_2_. Tissue was homogenized with 3 mm steel beads for 3 min using a HARBIL 5G-HD paint shaker (Fluid Management Inc. Wheeling, IL, USA). Genomic DNA was extracted from the homogenized samples using an urea-based method (Leach et al., 2016) and PCR amplified as described above. Primers were designed to amplify 0.5 kb-1 kb regions encompassing the sgRNA target site (**Supplementary Table 1**). PCR products were run on agarose gels to confirm successful amplification, and the remainder of the reaction was purified using either a QIAquick PCR purification kit (QIAGEN) or ExoSAP-IT (Thermo Fisher). Purified PCR products were submitted for Sanger sequencing with the TIDE protocol (Eurofins Genomics) using the forward primers. The resulting reads were analyzed for editing frequencies using the CRISPR ICE Analysis tool V3.0 (Conant et al., 2022). Somatic editing frequencies were calculated by averaging the percent mutagenesis observed in 4–5 T0 plants at 14 dpi. Allelic states of progeny were categorized based on theoretical expectations (25%, 50%, 75% or 100% per locus) with a ±7% buffer to account for technical noise. Samples were classified as mono-allelic (18-32%), bi-allelic (43-57%), tri-allelic (68-82%) or tetra-allelic (>90%).

## Results

### Establishing groundcherry lines for virus-induced gene editing

Before pursuing VIGE in groundcherry, we first evaluated how well Tobacco rattle virus (TRV) infects this species. To do this, we used a version of TRV2 that expresses the fluorescent protein AmCyan from *Anemonia majano* (Henderson and Remington, 2005) (**Supplementary Figure 1A**). This protein absorbs light in the blue spectrum (440–460 nm) and emits light in the cyan range (500–560 nm). TRV1 and TRV2-AmCyan were delivered to ground cherry seedlings through leaf infiltration. As the plants grew, high levels of fluorescence were observed throughout the plant, and this fluorescence persisted until fruit set (**Supplementary Figures 1B, C**). Because AmCyan was observed in distal, non-infiltrated tissue, this indicates its expression was due to TRV replication and systemic movement rather than localized transcription from the T-DNA delivered by the *Agrobacterium* inoculum. Thus, the systemic presence of the reporter confirms that *TRV* is a suitable viral vector for testing VIGE in groundcherry and that genome editing observed in distal tissue should be virus-mediated.

Because TRV cannot stably replicate large cargo, such as Cas9 from *Streptococcus pyogenes* (4.2 kb), we generated Cas9-expressing lines of groundcherry by *Agrobacterium*-mediated transformation. The T-DNAs used for transformation expressed Cas9 from a 35S promoter. A total of 31 independent transgenic lines were recovered through tissue culture, of which 27 tested positive for Cas9 by PCR. T1 seeds were collected from all 27 lines, and four lines were randomly selected for further analysis. Segregation of Cas9 was assessed by PCR in 9–13 T2 plants from each of the four lines. In two lines, Cas9 was transmitted to all assessed T2 progeny (**Supplementary Figure 2**), and these lines were selected for evaluation of Cas9 expression by immunoblot analysis. Among these, progeny of line pTC232–2 expressed Cas9, as indicated by a prominent band at approximately 158 kDa on immunoblots (**Supplementary Figure 3**).

In addition to immunoblotting, Cas9 activity was also assessed among the four lines by testing for gene editing. Seedlings were infiltrated with three *Agrobacterium* strains: one expressing TRV1 and two strains each expressing one of two sgRNAs (designated sgRNA1 and sgRNA2). These sgRNAs target the *P. grisea PHYTOENE DESATURASE* (*PDS*) gene, with target sites located in either in the first or fifth exon (**Supplementary Figure 4A**). To enhance sgRNA mobility and editing efficiency, the sgRNAs were augmented with a sequence derived from *Arabidopsis thaliana FlOWERING LOCUS T* (*FT*), which has been shown to promote mobility of sgRNAs between cells and thereby to increase gene editing frequencies (Ellison et al., 2020; Jackson and Hong, 2012; Nagalakshmi et al., 2022). At three days post infection (dpi) the infiltrated tissue was harvested, and gene editing was evaluated by PCR-amplifying the *PDS* locus using primers flanking both sgRNA target sites. The expected 5.1kb amplicon was detected in all samples; however, the infected pTC232–2 plant also displayed a smaller 0.6 kb band (**Supplementary Figure 4B**). Sanger DNA sequencing of the 0.6 kb amplicon revealed that it arose due to a deletion of genomic DNA sequences between the two sgRNA target sites (**Supplementary Figure 4C**). Based on the immunoblotting results and the in planta assessment of gene editing, the pTC232–2 line and sgRNA2 were selected to more thoroughly investigate somatic cell gene editing and to determine whether heritable gene edits could be recovered.

### Virus-mediated somatic cell gene editing

**Figure 1:**
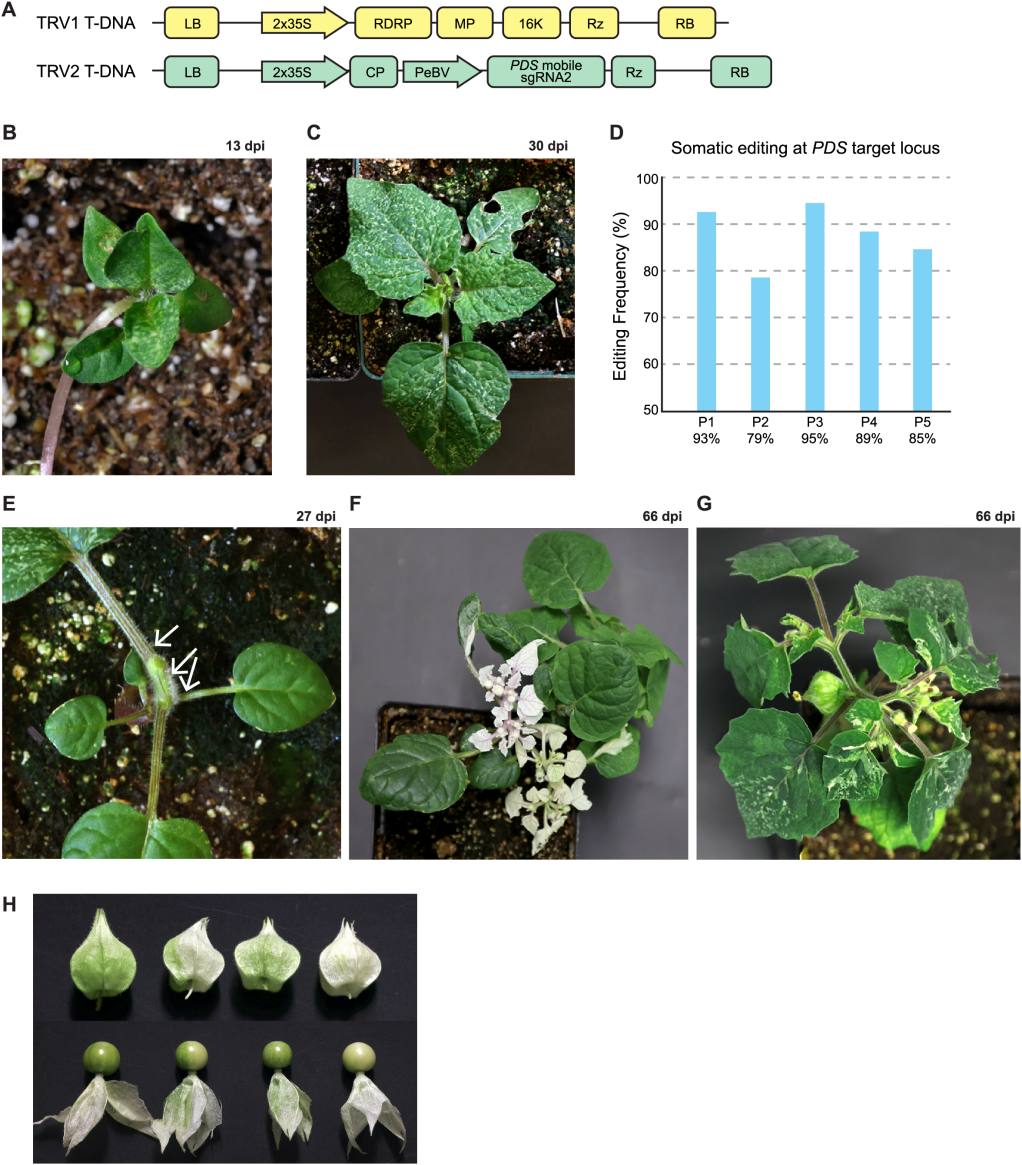
Somatic and heritable gene editing with trimming. **(A)** TRV1 and TRV2 were delivered to plant cells by agroinfiltration. The left and right borders of the T-DNA (LB and RB, resectively) are shown. The double 35S promoter drives transcription of TRV1 and TRV2. initiating viral infection. TRV1 encodes the RNA-dependent RNA polymerase (RdRP), movement protein (MP), the 16K suppressor of RNA silencing, and a terminating nbozyme (Rz). TRV2 encodes the coat protein (CP) and contains the Pea early browning virus (PeBV) subgenomic promoter, which drives expression of the mobile PDS sgRNA2, followed by a terminating nbozyme (Rz). **(B, C)** A seedling infected with TRV expressing the PDS mobile sgRNA exhibits photobleaching speckling in systemic leaves at 13 days post infection (dpi) **(B)** and 30 dpi **(C, D)** Somatic editing frequencies at the PDS sgRNA2 target site across five plants. Editing frequencies were estimated using the Synthego ICE analysis tool. **(E)** A plant with speckled leaves was trimmed at the apical and axillary meristems to induce new shoot formation; trimming sites are indicated by arrows. **(F)** Representative image of a trimmed plant at 66 dpi showing completely photobleached branches emerging from trimming sites. **(G)** A non-trimmed plant at 66 dpi displays speckled branches and lacks fully photobleached branches. **(H)** Fruit from plants infected with PDS sgRNA2 display a range of photobleaching phenotypes in the husks. Partially photobleached fruits are commonly observed in untrimmed plants, whereas completely white fruits are frequently observed in trimmed plants.

To assess somatic cell gene editing, pTC232–2 seedlings were infiltrated with *Agrobacterium* strains delivering TRV1 and TRV2-sgRNA2 (**Figure 1A**). Infiltrated plants began developing photobleached spots on the upper leaves at approximately 13 dpi, suggestive of *PDS* editing (**Figure 1B**). This phenotype became more pronounced as the plants matured, with newly emerging leaves displaying extensive speckling by 30 dpi (**Figure 1C**). The sgRNA2 target site was PCR-amplified from genomic DNA isolated from these leaves, and Sanger DNA sequencing revealed editing frequencies of approximately 80-95% across all infected plants (**Figure 1D**).

We previously demonstrated that pruning tomato plants undergoing VIGE can induce formation of new shoots from edited somatic cells (Liu et al., 2024). Oftentimes these newly induced shoots have fixed gene edits that can be transmitted to the next generation. To test whether this approach is effective in groundcherry, apical and lateral meristems were removed from plants 3–4 weeks post infection (**Figure 1E**) as described in Liu et al., 2024. Pruned plants typically developed multiple branches at the trim site, many of which exhibited had photobleached leaves (**Figure 1F**). In contrast, unpruned plants displayed only speckled leaves at the same stage of development (**Figure 1G**). However, when unpruned plants were grown under higher daytime temperatures (i.e. 26 °C vs. 22 °C), they often produced fully photobleached branches (**Supplementary Figure 5**).

As plants flowered and set fruit, sepals and fruit husks showed varying degrees of photobleaching, ranging from speckled to completely white. Fruits emerging from photobleached branches were predominantly white, whereas speckled or partially white fruits were produced on plants with similarly patterned leaves. Fruit and husk phenotypes were also closely correlated, suggesting a strong association between husk pigmentation and fruit color (**Figure 1H**).

### Virus-mediated heritable gene editing

**Figure 2:**
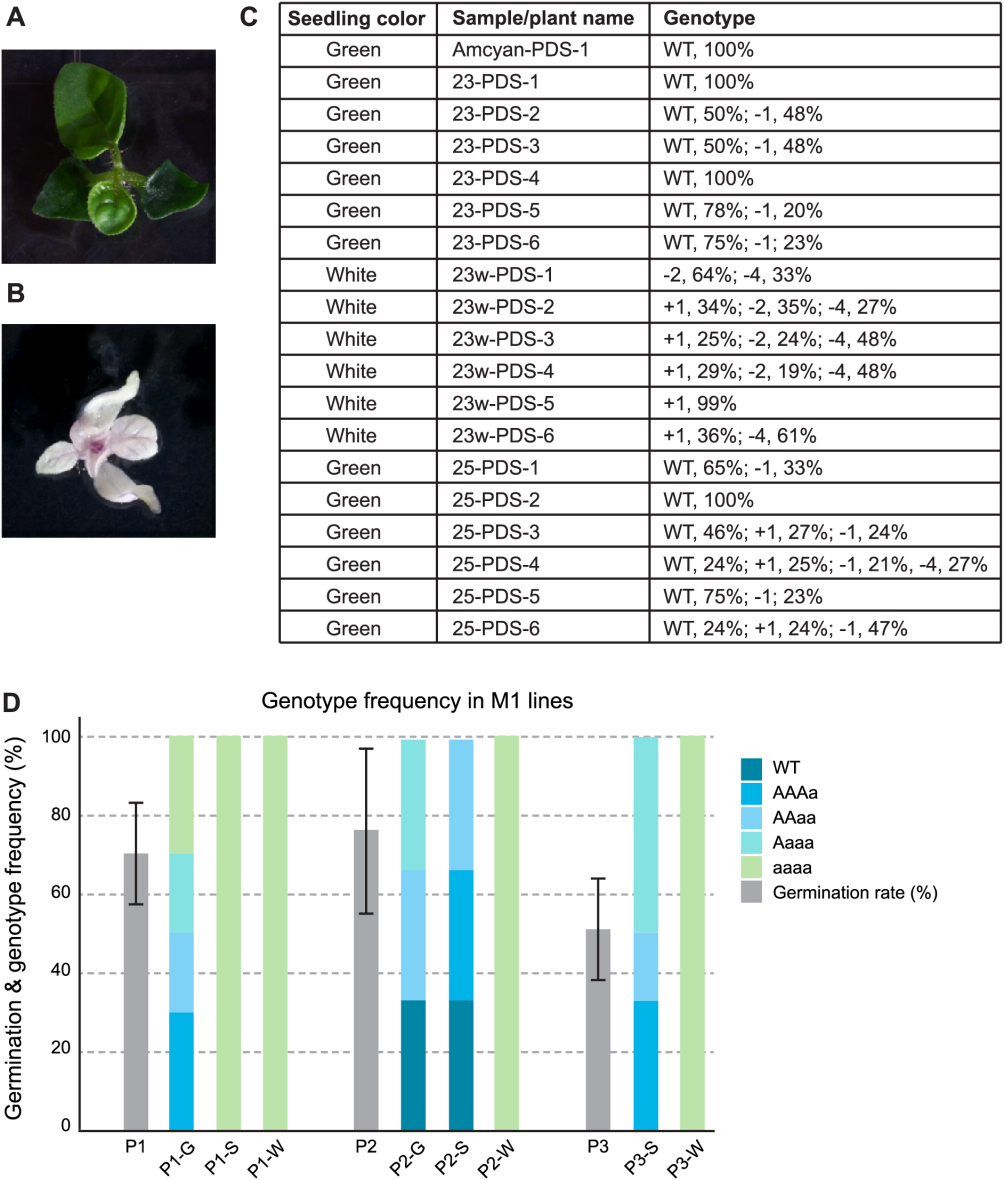
Heritable gene editing at PDS. **(A, B)** Representative green **(A)** and white **(B)** seedlings derived from fruit that was partially or completely photobleached, respectively. **(C)** Representative genotyping results showing that green seedlings carried either a wild-type (WT) allele or small in-frame mutations (e.g., ±3-bp indels), whereas all alleles recovered from white seedlings contained inactivating frameshift mutations. **(D)** Genotype frequencies in M, progeny from three independent replicates (P1-P3) infected with PDS sgRNA2. Fruit color from each parent plant is indicated adjacent to the plant number (G, green; S, speckled; W, white). Some plants, such as P3, did not produce green fruit, with all fruit being either speckled or white. Germination frequencies (40-100%) are shown for each replicate. The PDS wild-type allele is denoted as "A," whereas mutant alleles are denoted as "a." Samples were classified as mono-allelic (18-32% indels), bi-allelic (43-57% indels), tri-allelic (68-82% indels), or tetra-allelic (>90% indels). All seedlings assayed fell within these ranges.

To assess whether gene edits could be transmitted to the next generation, we selected five infected plants exhibiting a range of photobleaching phenotypes, and three fruits were collected from each plant. The selected fruits displayed varying degrees of photobleaching, ranging from green to completely white (**Figure 1H**). Seeds from these fruits were germinated on solid medium in Petri dishes, and germination rate, M1 seedling color, and gene editing at the target locus were evaluated. Seeds derived from completely white fruit produced all-white seedlings, whereas seeds from speckled and green fruit yielded a mixture of green and white seedlings (**Figures 2A, B**). Three to six seedlings from each fruit were assessed for mutations at the sgRNA2 target site (**Figure 2C**). All alleles in white seedlings had frameshift mutations at the PDS target, whereas green seedlings had at least one wild-type allele.

Because *P. grisea* is a diploid (2n = 24), we anticipated recovering no more than two distinct alleles per progeny. However, sequencing data revealed many progeny had up to four distinct alleles (e.g. plant 25-PDS-4 in **Figures 2C, D**). In other cases (e.g. plants 23-PDS-5 and 23-PDS-6), sequencing reads were present at approximately a 3:1 ratio of wild-type to mutant alleles, a pattern inconsistent with diploidy. We considered that perhaps line pTC232–2 is a tetraploid resulting from a genome duplication that took place during regeneration through tissue culture. Indeed, Swartwood et al. (2025) showed that in *P. grisea*, ploidy variation can originate at the plant regeneration phase (Swartwood et al., 2025). To assess our lines, flow cytometry was performed on nuclei generated from pTC232–2 and a wild-type (i.e. non-transformed) plant (**Supplementary Figure 6**). The fluorescence of nuclei from the wild-type and pTC232–2 line – in both individual and mixed runs – confirmed that pTC232–2 is a tetraploid.

### Targeting the domestication gene, *CLAVATA1*

**Figure 3:**
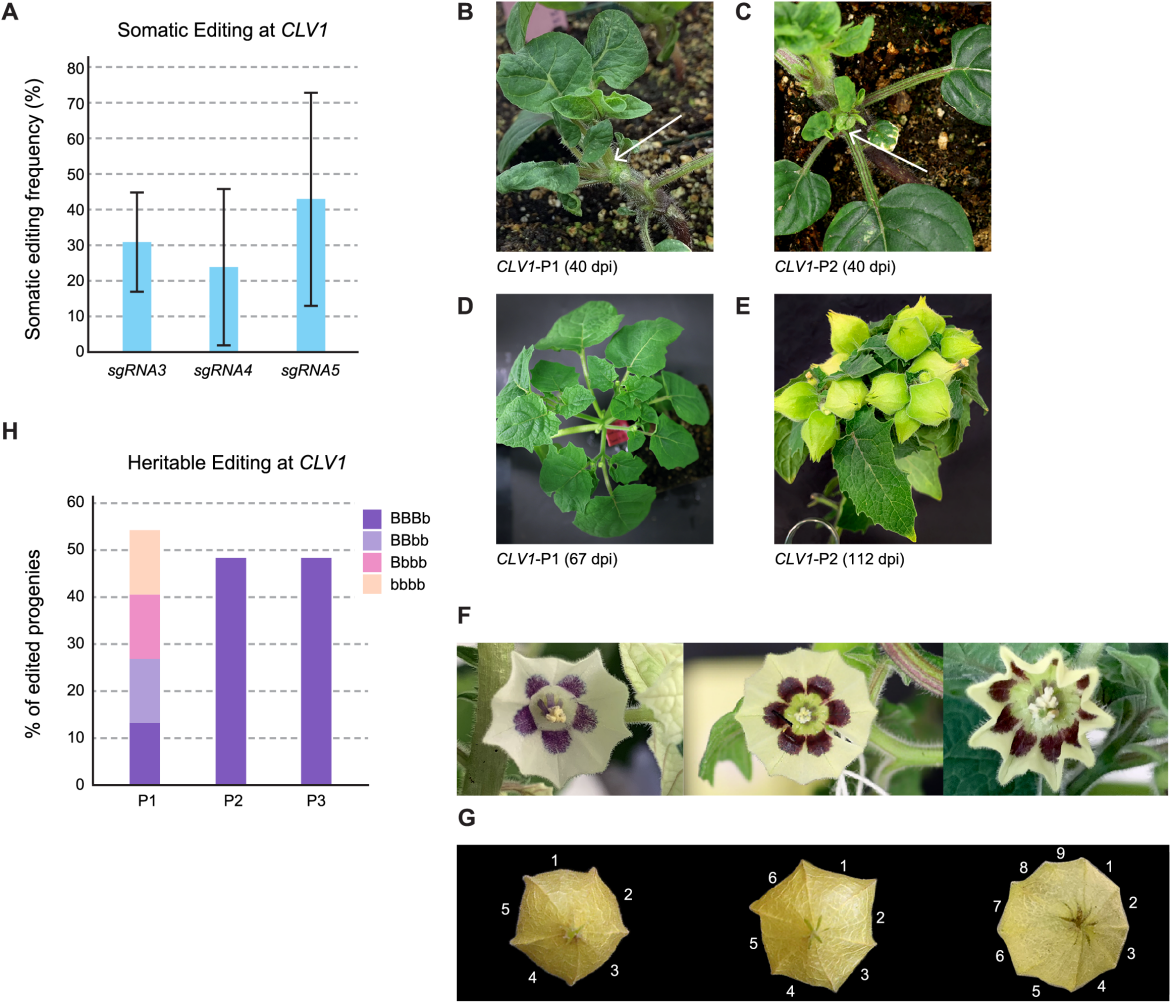
Editing of the domestication gene CLV1. **(A)** Somatic editing frequencies of three sgRNAs targeting CLV1. Editing frequencies were calculated across multiple infected plants. Bars represent the mean editing frequency calculated from four independent TO plants per construct (n=4). Error bars indicate the standard deviation (SD), reflecting the variability in editing frequencies between individual plants. **(B, C)** Plants infected with TRV expressing CLV1 sgRNA5 at 40 dpi, exhibiting somatic editing frequencies of 48% and 28%, respectively; images show recovered growth 1.5 months post-pruning. **(D, E)** The same individuals from **(B, C)** displaying healthy vegetative growth and increased lateral branching 2.5 months post-pruning. **(F)** Representative flowers from uninfected Cas9 plants (negative control) displaying five petals, compared with flowers from plants infected with TRV-CLV1 sgRNA5, which exhibit increased petal numbers (6-9 petals). **(G)** Fruit derived from the flowers shown in **(F)** displaying increased locule numbers (6-9 locules). **(H)** Heritable editing frequencies in progeny (8-16 per plant) from three independently infected parent plants, demonstrating heritable editing frequencies ranging from 50-57%. Wild-type CLV1 alleles are designated as "B," whereas mutant alleles are designated as "b." Plant 1 (CLV1-P1), which exhibited the highest somatic editing frequency (49%), produced progeny with diverse genotypes, including homozygous mutant (bbbb) individuals. Plants 2 and 3 produced progeny carrying only mono-allelic mutations (BBBb).

To demonstrate that virus-induced gene editing could create agriculturally relevant traits in groundcherry, we targeted *CLAVATA1* (*CLV1*), which when inactivated, is known to increase fruit size and mass in tomato and groundcherry (Lemmon et al., 2018; Xu et al., 2015). pTC232–2 seedlings were agroinfiltrated with TRV vectors expressing one of three mobile sgRNAs targeting the first exon of *CLV1* (**Supplementary Table 2**). Somatic editing frequencies in T0 seedlings (n=4, 14 dpi) ranged from 24-73% across the three sgRNA target sites, with the highest mutagenesis frequencies achieved using sgRNA5 (**Figure 3A**). Three weeks post infection, all plants were trimmed to remove apical and axillary meristems, and newly emerged shoots were genotyped. Plants lacking detectable somatic editing grew normally; however, shoots with high frequencies of mutagenesis displayed pronounced developmental abnormalities, including deformed leaves and many branches (**Figures 3B, C**). As these shoots matured, most developed a compact growth habit with tightly clustered fruits (**Figures 3D, E**). Flowers from edited branches had 6–9 petals in contrast to the 5 petals found in wild-type plants (**Figure 3F**); most fruit had 3–4 locules (**Figure 3G**), compared to 2 locules typical of the wild-type.

To assess heritable editing at *CLV1*, we selected plants with abnormal growth habits and fruits with more than two locules. Analysis of the sgRNA5 target site at *CLV1* in 8–16 progeny from three such plants revealed approximately 60% heritable editing (**Figure 3H**). The most common genotype observed was a mono-allelic mutation (**Supplementary Figure 7**), though progeny from Plant 1 exhibited a mix of mono-, bi-, tri- and tetra-allelic mutations (**Figure 3H**).

### Duplex editing with a single TRV2 vector

We have previously shown that TRV2 vectors can be engineered to express more than one sgRNA to enable multiplexed gene editing following viral infection (Ellison et al., 2020). To test this in groundcherry, we constructed a TRV2 vector expressing both *PDS* sgRNA2 and *CLV1* sgRNA3, each fused with their respective *FT* mobility sequences. This vector was delivered along with TRV1 to pTC232–2 plants via agroinfiltration, and phenotypic changes were monitored prior to genotypic analysis. Among the infected plants (n=10), approximately 50% exhibited a speckling phenotype and approximately 20% showed flowers with more than five petals (**Supplementary Figure 8A**). Somatic editing at both target loci was detected in ~30% of the plants, with editing frequencies ranging from 56-92% at the *PDS* target and 5-25% at the *CLV1* locus (**Supplementary Figure 8B**).

## Discussion

In this study, we demonstrate that Tobacco rattle virus (TRV)-mediated delivery of mobile sgRNAs enables highly efficient somatic and heritable genome editing in groundcherry (*Physalis grisea*). We define ‘high efficiency’ as the consistent ability of the TRV vector to move systemically and facilitate heritable editing. By combining viral delivery with constitutive Cas9 expression, we achieved robust editing at multiple loci, including the visual marker gene *PHYTOENE DESATURASE* (*PDS*) and the domestication gene *CLAVATA1* (*CLV1*). These findings expand the range of dicot species amenable to virus-induced gene editing (VIGE) and establish groundcherry as a tractable system for rapid genetic improvement without repeated tissue culture-related methodology.

A key outcome of this work is the consistently high frequency of heritable editing achieved using TRV. All plants infected with *PDS*-targeting mobile sgRNAs produced seeds carrying *PDS* mutations, with photobleached progeny recovered from more than 30 independent plants. This result is consistent with earlier observations that TRV can access germline or germline-adjacent tissues, in contrast to many plant viruses – particularly those infecting monocots – that are excluded from meristems. Interestingly, in experiments targeting *PDS*, higher frequencies of editing were observed at higher temperatures, as evidenced by the production of large numbers of completely photobleached shoots. This contrasts with our observation in *N. benthamiana* and others in groundcherry that lower temperatures are more conducive to viral infectivity and amplification of the viral vectors (Ellison et al., 2020; Zhang et al., 2024). It may be that an increase in Cas9 activity at higher temperatures offsets the negative impact of higher temperatures on viral replication. Regardless, the ability of TRV to induce heritable edits across multiple solanaceous species highlights the broad utility of this virus as a gene delivery platform for in planta genome editing.

An unexpected but informative outcome of this study was the discovery that the primary Cas9-expressing line used for most experiments was tetraploid. In a recent report, it was shown that ploidy variation in *P. grisea* tissue culture-derived lines occurs during the plant regeneration phase (Swartwood et al., 2025). Despite the increased allelic complexity associated with tetraploidy, we observed high somatic and heritable editing efficiencies, with progeny exhibiting mono-, bi-, tri-, and tetra-allelic mutations. These results indicate that VIGE remains highly effective even when multiple alleles must be simultaneously targeted, underscoring the robustness of this approach and its potential applicability to polyploid crop species. We observed notable variability in the specific allelic compositions among progeny from independently infected parental plants. This variation likely reflects the stochastic nature of virus-mediated systems, where the timing of viral entry into meristematic tissues and the rate of systemic spread can vary between individuals. Because the final heritable editing outcome is determined by the specific developmental window during which the virus accesses germline-adjacent cells, differences in the speed of infection or the plant’s developmental stage at the time of viral establishment can lead to diverse mutational profiles in the next generation.

While *PDS* provides a convenient visual marker for validating gene editing, practical crop improvement requires targeting genes with subtler phenotypes. Editing of *CLV1* resulted in phenotypes consistent with disrupted floral meristem regulation, including increased branching, flowers with additional organ whorls, and fruits with increased locule number. Although we did not quantitatively measure fruit size or yield, the observed phenotypes align with previous studies in groundcherry and tomato and suggest increased fruit production results from meristem overgrowth (Lemmon et al., 2018). Importantly, progeny derived from edited plants exhibited a range of allelic combinations, providing material to assess gene dosage effects on fruit traits. This capacity to generate multiallelic series is particularly valuable for creating quantitative variation, including through the editing of regulatory elements.

For targets such as *CLV1*, where mutant phenotypes are less visually striking than *PDS*, we found that trimming infected plants to induce *de novo* shoot formation enhanced the recovery of shoots with fixed edits. Newly regenerated shoots frequently displayed uniform mutant phenotypes, indicating their origin from edited somatic cells. Although trimming was not required to achieve heritable editing at the *PDS* locus, it proved advantageous for recovering stable edits at loci with more subtle phenotypic effects. This strategy complements recent approaches that exploit the plant’s regenerative capacity to bypass conventional tissue culture while enabling efficient genome modification (Maher et al., 2020).

To further streamline the recovery of edits with non-visual phenotypes and to minimize genotyping burdens, inclusion of a fluorescent reporter, such as AmCyan, in the TRV2 vector could serve as a proxy for viral titer and systemic spread. Selective harvesting of seed derived from tissues with high reporter expression might increase the probability of recovering heritable edits. For plant species or genotypes with reduced regenerative capacity, morphogenic regulators (e.g., *BBM*, *WUS*, *GRF-GIF* chimeras) could be co-delivered, or transgenic plants could be used that express these morphogenic regulators under inducible promoters. Combining such enrichment strategies with VIGE’s inherent ability to generate diverse alleles holds promise for creating genetic diversity that contributes to complex quantitative traits where direct visualization of a phenotype is not possible.

VIGE offers a clear advantage in speed compared to *Agrobacterium*-mediated transformation. While conventional transformation in groundcherry typically requires several months to recover regenerated plants, our in planta approach produced edited seeds within 3–4 months, closely matching the natural life cycle of the species. The modest delay observed in some experiments was primarily attributable to trimming, which was selectively employed to enhance fixation of edits at non-*PDS* loci. Notably, trimming was unnecessary for achieving heritable editing at *PDS*, highlighting the flexibility of the system depending on experimental goals.

A major advantage of the viral-mediated strategy described here is that tissue culture is required only once to generate the initial Cas9-expressing lines, which can subsequently be reused to generate diverse genetic variation. Nevertheless, tissue culture-associated effects, including changes in ploidy, remain a limitation. Recent studies have shown that TRV can be engineered to deliver both guide RNAs and compact nucleases, enabling genome editing without stable transgene integration (Hu et al., 2025; Weiss et al., 2025). Applying such tissue culture–free approaches in groundcherry would eliminate the need for nuclease-expressing transgenic intermediates, with potential regulatory benefits in jurisdictions where transgenic plants are subject to additional oversight.

Finally, we demonstrate that multiplexed editing is feasible in groundcherry using a single TRV vector carrying multiple mobile sgRNAs. Simultaneous targeting of *PDS* and *CLV1* resulted in somatic editing at both loci, illustrating the potential of this approach for stacking desirable alleles in a single generation. The observed reduction in *CLV1* editing efficiency during duplex targeting likely reflects either sgRNA competition for Cas9 loading, locus-specific chromatin accessibility, or inherent differences in sgRNA efficacy, observations consistent with locus-dependent effects in other multiplex CRISPR systems (Ma et al., 2016; Weiss et al., 2022; Uranga et al., 2021). As interest grows in *de novo* domestication and improvement of underutilized crops, viral-mediated genome editing provides a powerful means to overcome persistent bottlenecks in transformation and regeneration. Together, our results position VIGE as a scalable and versatile platform for accelerating genetic improvement in groundcherry and other neglected and underutilized crops.

## Data Availability

The original contributions presented in the study are included in the article/**Supplementary Material**. Further inquiries can be directed to the corresponding author.
