# Supplementary material for: Virus-mediated, heritable gene editing in groundcherry (Physalis grisea)
Source: Front Plant Sci. 2026 Mar 20;17:1794888. doi: 10.3389/fpls.2026.1794888 (PMC13047112; doi:10.3389/fpls.2026.1794888)
Supplement: Supplementary file 1 [file Image1.pdf]

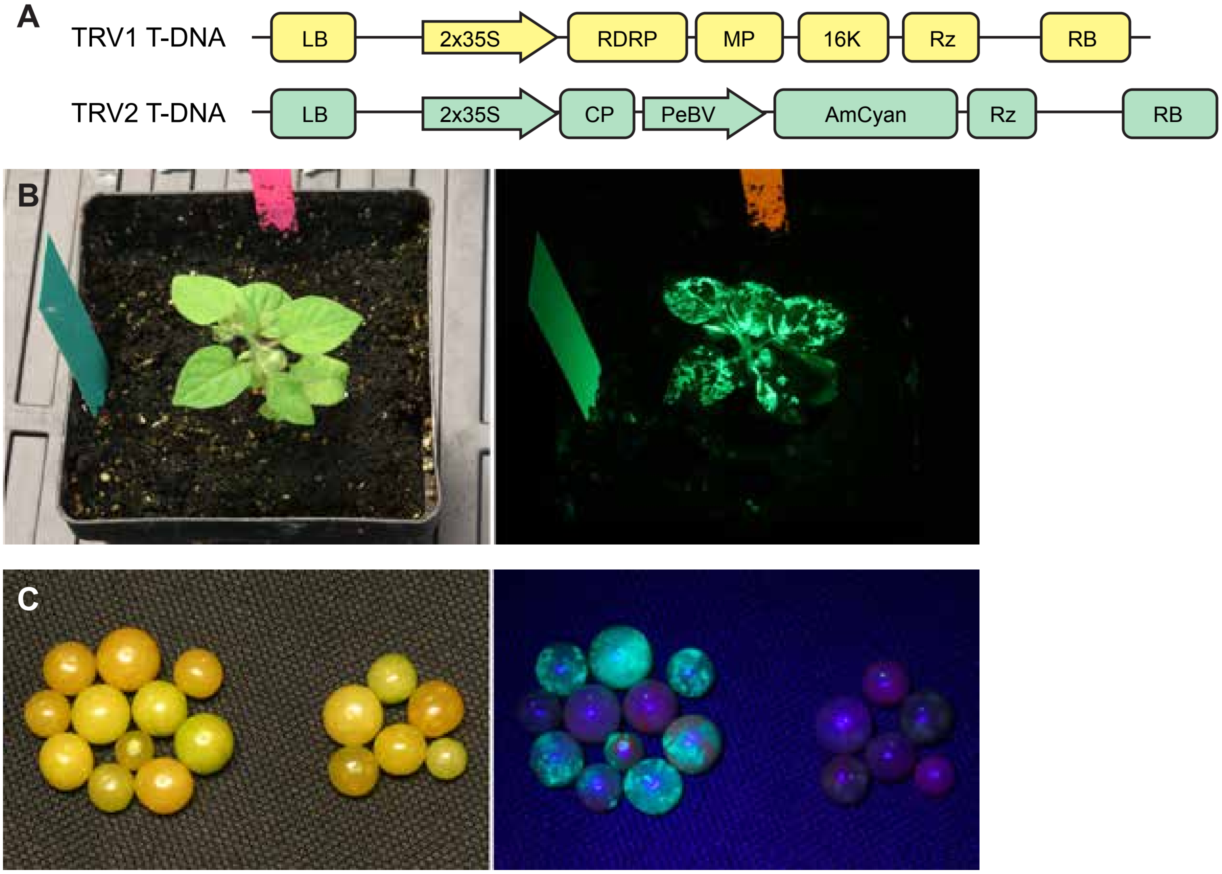

Supplementary Figure 1. Infection of groundcherry with TRV. (A) A TRV2 construct expressing AmCyan from the Pea early browning virus subgenomic promoter was generated. TRV1 and TRV2-AmCyan were delivered to wild-type groundcherry seedlings by agroinfiltration. **(B)** Representative images of infected plants under white light (left) and blue light (right). **(C)** Representative images of fruit harvested from infected and uninfected plants under white light (left) and blue light (right).
