# Supplementary material for: Virus-mediated, heritable gene editing in groundcherry (Physalis grisea)
Source: Front Plant Sci. 2026 Mar 20;17:1794888. doi: 10.3389/fpls.2026.1794888 (PMC13047112; doi:10.3389/fpls.2026.1794888)
Supplement: Supplementary file 2 [file Image2.pdf]

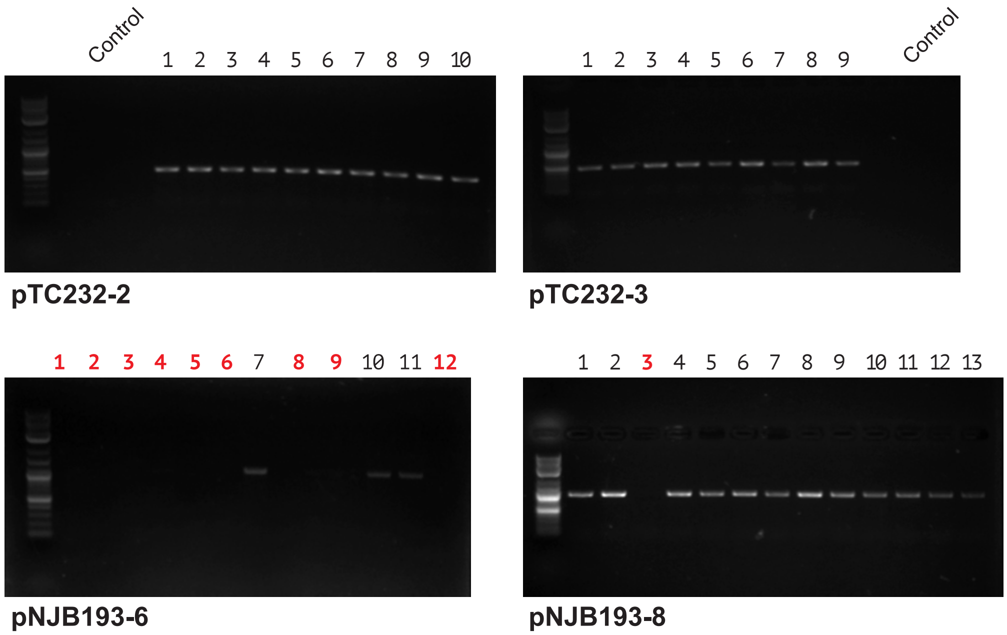

Supplementary Figure 2. PCR analysis of Cas9 segregation in T_2 lines. The presence of the Cas9 transgene was assessed in 9 to 13 T_2 plants derived from four Cas9-positive T_1 lines (pTC232-2, pTC232-3, pNJB193-8, and pNJB193-6). Amplification of a 489-bp fragment from the pTC lines and a 1,031-bp fragment from the pNJB lines indicates the presence of Cas9. Lanes labeled in red indicate T_2 plants that tested negative for the Cas9 transgene.
