# Supplementary material for: Virus-mediated, heritable gene editing in groundcherry (Physalis grisea)
Source: Front Plant Sci. 2026 Mar 20;17:1794888. doi: 10.3389/fpls.2026.1794888 (PMC13047112; doi:10.3389/fpls.2026.1794888)
Supplement: Supplementary file 3 [file Image3.pdf]

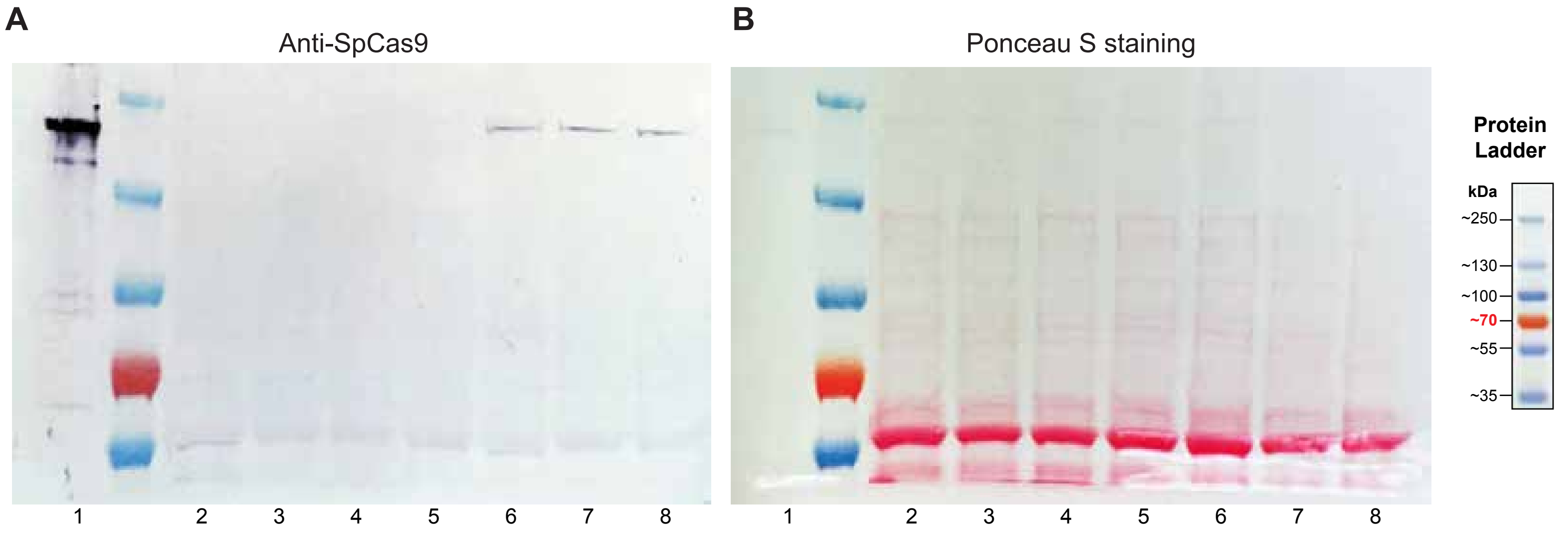

Supplementary Figure 3. Immunoblot analysis of Cas9 in T_2 lines. **(A)** Immunoblot analysis of soluble protein extracts from two-week-old T_2 groundcherry plants using anti-Cas9 antibodies. Lane 1, purified Cas9 protein (positive control); lanes 2 and 3, extracts from wild-type groundcherry (negative control); lane 4, pNJB193-6-10; lane 5, pNJB193-8-3; lane 6, pTC232-2-2; lane 7, pTC232-2-4; and lane 8, pTC232-2-16. **(B)** Ponceau S staining of the nitrocellulose membrane used in **(A)** showing comparable protein loading across lanes. The inset indicates the molecular mass (kDa) of the protein standards.
