# Supplementary material for: Virus-mediated, heritable gene editing in groundcherry (Physalis grisea)
Source: Front Plant Sci. 2026 Mar 20;17:1794888. doi: 10.3389/fpls.2026.1794888 (PMC13047112; doi:10.3389/fpls.2026.1794888)
Supplement: Supplementary file 4 [file Image4.pdf]

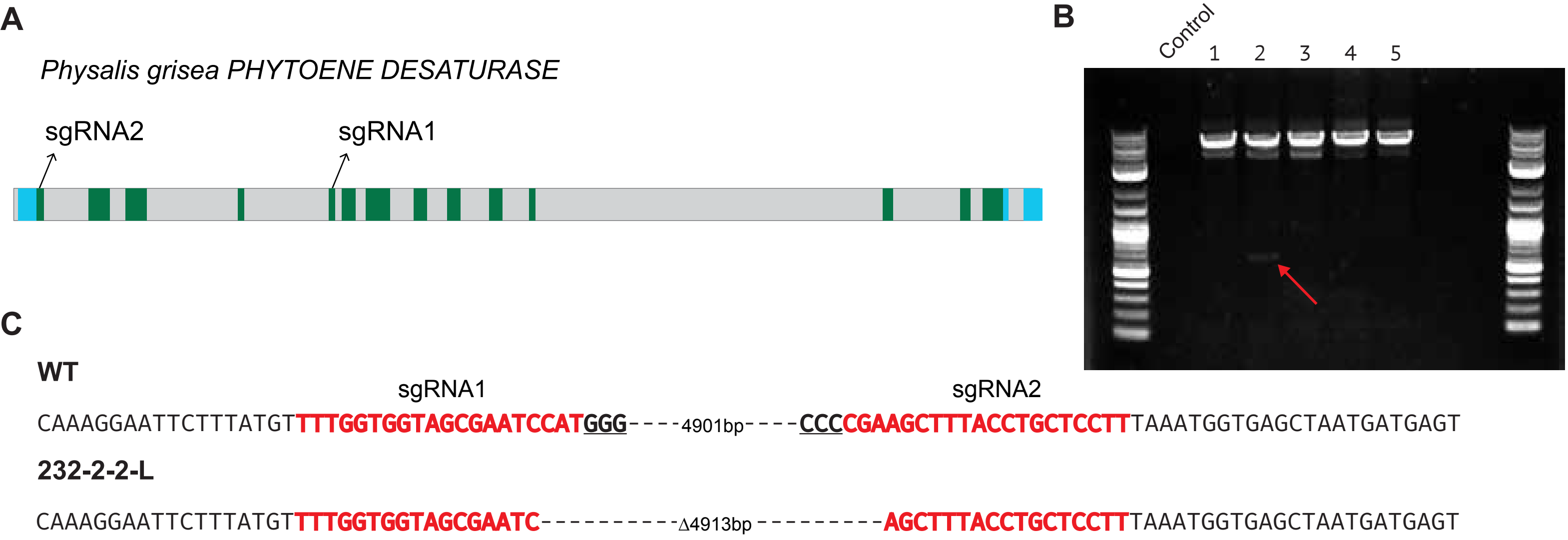

Supplementary Figure 4. Analysis of gene editing in T_2 lines. (A) Gene model of the *Physalis grisea PHYTOENE DESATURASE (PDS)* gene. Green boxes indicate exons while blue boxes represent 5' and 3' UTRs; sgRNA target sites are located in the first and fifth exons. **(B)** Agarose gel image of PCR amplicons spanning the two sgRNA target sites (~5 kb). Lane 1, pTC232-2-2 not infected with TRV. Lanes 2 to 5 represent different Cas9 transgenic lines infected with TRV1 and a mixture of TRV2 vectors expressing sgRNA1 and sgRNA2: lane 2, pTC232-2-2; lane 3, pTC232-3-6; lane 4, pNJB193-8-11; and lane 5, pNJB193-6-8. The arrow indicates the PCR product resulting from deletion between the two sgRNA target sites in pTC232-2-2. **(C)** DNA sequencing of the band indicated in **(B)** confirms a precise deletion between the two sgRNA target sites. sgRNA target sequences are shown in bold, and the PAM sequences are underlined.
