# Supplementary material for: Virus-mediated, heritable gene editing in groundcherry (Physalis grisea)
Source: Front Plant Sci. 2026 Mar 20;17:1794888. doi: 10.3389/fpls.2026.1794888 (PMC13047112; doi:10.3389/fpls.2026.1794888)
Supplement: Supplementary file 5 [file Image5.pdf]

72 dpi **90 dpi**

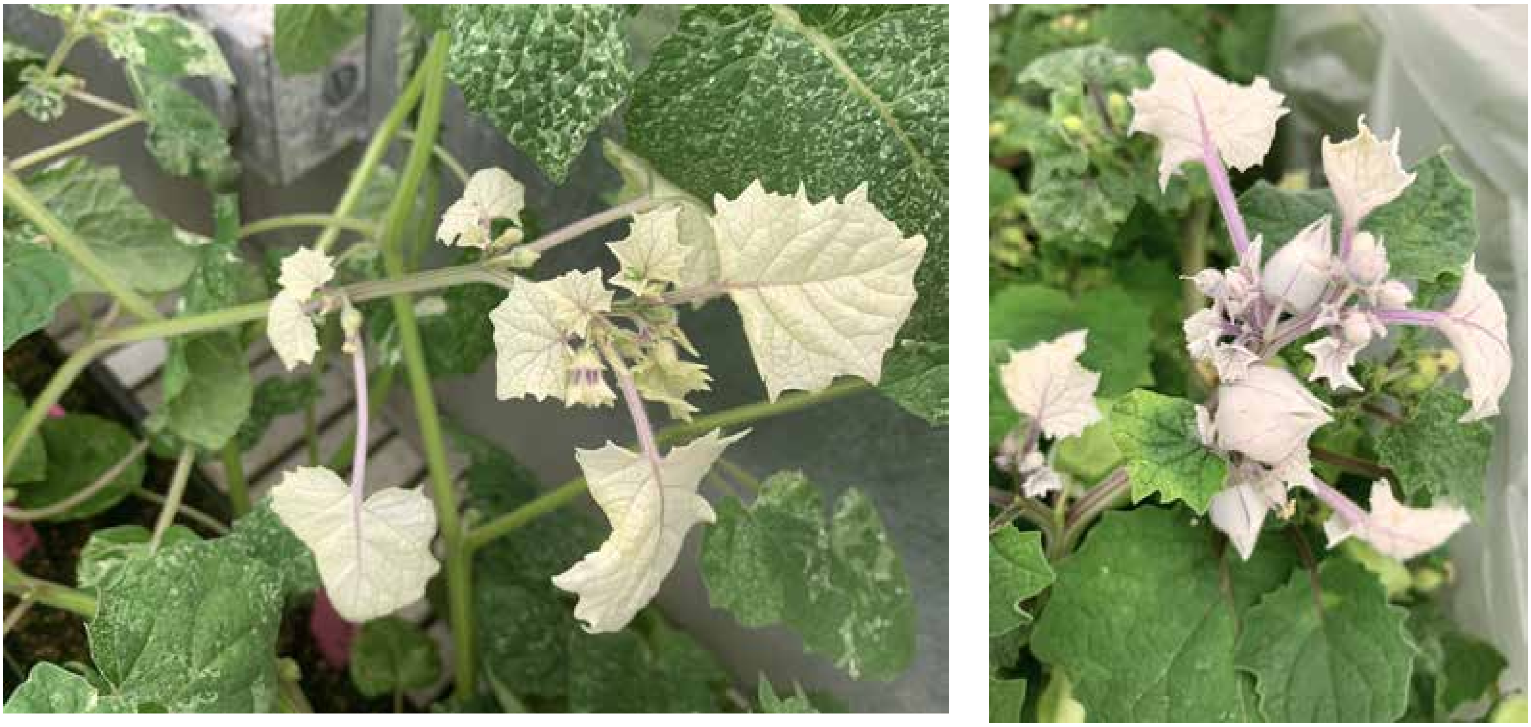

Supplementary Figure 5. Growth at elevated temperature induces photobleaching in TRV-infected plants. Plants were infected with TRV1 and TRV2-sgRNA2 and grown at 26°C. Completely photobleached shoots, inflorescences, and fruit are shown at 72 and 90 days post infection (dpi). Plants were not subjected to trimming.
