# Supplementary material for: Virus-mediated, heritable gene editing in groundcherry (Physalis grisea)
Source: Front Plant Sci. 2026 Mar 20;17:1794888. doi: 10.3389/fpls.2026.1794888 (PMC13047112; doi:10.3389/fpls.2026.1794888)
Supplement: Supplementary file 6 [file Image6.pdf]

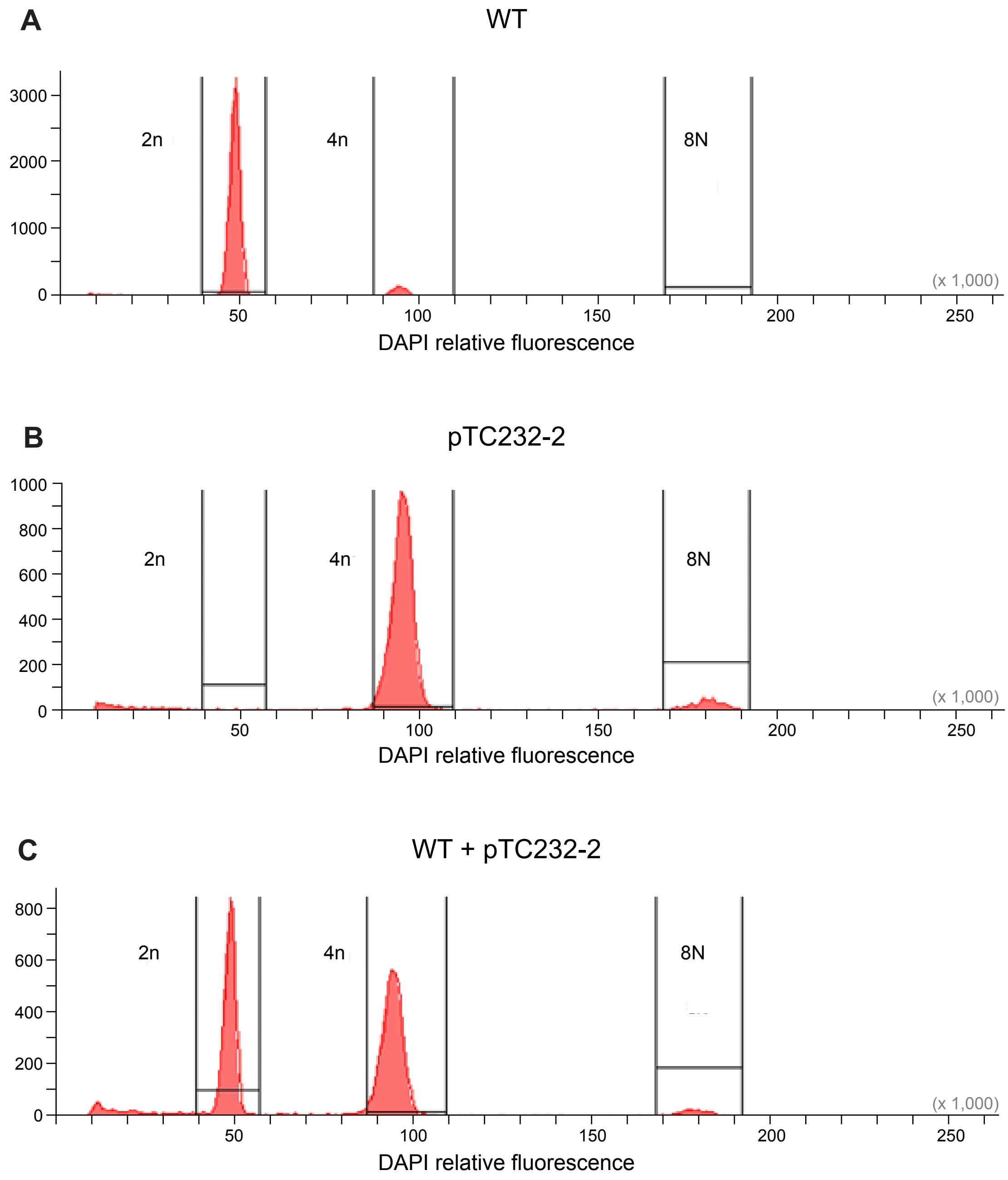

Supplementary Figure 6. Analysis of nuclei from pTC232-2 and wild-type groundcherry. **(A)** Relative DAPI fluorescence of nuclei from wild-type groundcherry. **(B)** Relative DAPI fluorescence of nuclei from the pTC232-2 line. **(C)** Relative DAPI fluorescence of a mixed population of nuclei from pTC232-2 and wild-type groundcherry.
