# Supplementary material for: Virus-mediated, heritable gene editing in groundcherry (Physalis grisea)
Source: Front Plant Sci. 2026 Mar 20;17:1794888. doi: 10.3389/fpls.2026.1794888 (PMC13047112; doi:10.3389/fpls.2026.1794888)
Supplement: Supplementary file 7 [file Image7.pdf]

A

CLV1 sgRNA3-P1-M3

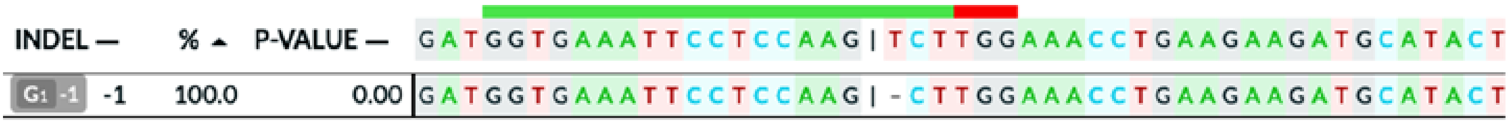

B

CLV1 sgRNA5-P2-M2

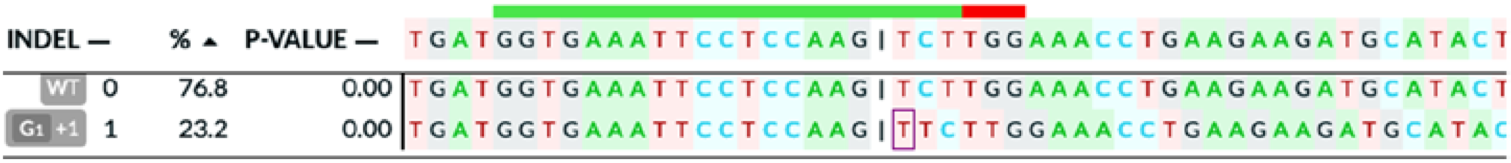

Supplementary Figure 7. Genotypes of representative progeny with mutations at the *CLV1* target site. (A) A progeny from plant 1 that is homozygous for a single–base pair deletion at the expected cut site of *CLV1* sgRNA3. **(B)** A progeny from plant 2 that is mono-allelic (~25%) for a single–base pair insertion at the expected cut site of *CLV1* sgRNA5.
