# Supplementary material for: Virus-mediated, heritable gene editing in groundcherry (Physalis grisea)
Source: Front Plant Sci. 2026 Mar 20;17:1794888. doi: 10.3389/fpls.2026.1794888 (PMC13047112; doi:10.3389/fpls.2026.1794888)
Supplement: Supplementary file 8 [file Image8.pdf]

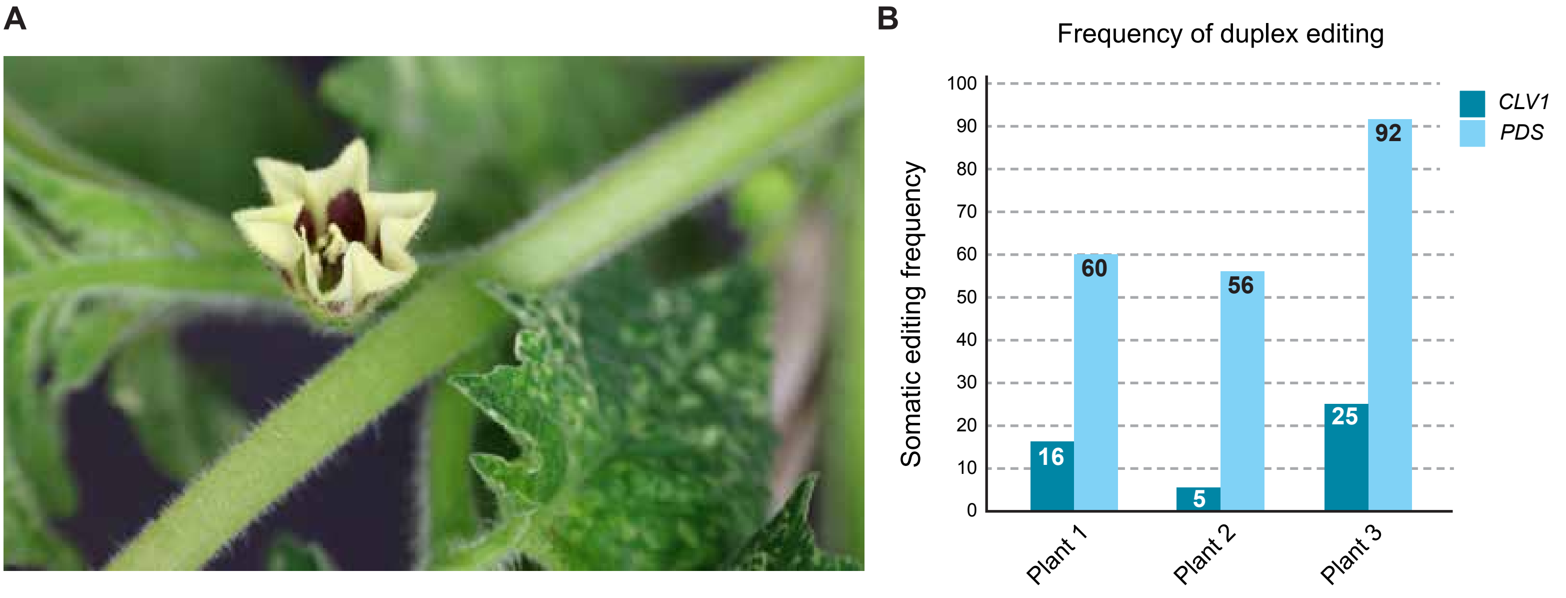

Supplementary Figure 8. Duplex editing at *CLV1* and *PDS*. (A) Representative plant infected with TRV2 expressing sgRNAs targeting *CLV1* and *PDS*. Photobleached leaves and a flower with six locules are shown, consistent with *CLV1* loss-of-function phenotypes. **(B)** Somatic gene editing frequencies at the *CLV1* and *PDS* target sites in three independent plants. Editing frequencies at the *PDS* locus were consistently higher than those at the *CLV1* locus across all plants tested.
