# Supplementary material for: Virus-mediated, heritable gene editing in groundcherry (Physalis grisea)
Source: Front Plant Sci. 2026 Mar 20;17:1794888. doi: 10.3389/fpls.2026.1794888 (PMC13047112; doi:10.3389/fpls.2026.1794888)
Supplement: Supplementary file 9 [file Table1.pdf]

Primers	Sequence (5'-3')	Description
oTC199	CCTCAAGTCTGATGGATTCGC	Forward primer for PCR amplification of SpCas9 from pTC232 lines
oCS1133	GACTGTGGCACGATATGATCAACATC	Reverse primer for PCR amplification of SpCas9 from pTC232 lines
oEE1616	GGGCTGTTATCACCGATGAG	Forward primer for amplification of SpCas9 from pNJB193 lines
oEE1617	TCCAGCGTATCCGTTCTTAG	Reverse primer for amplification of SpCas9 from pNJB193 lines
oRT491	TTGGAAATGTCACAGCTTGCT	Forward primer for amplification of the *CLV1* sgRNA3 target site & DNA sequencing
oRT492	ACCTGTAAGTCTGTTCACTTGGAGAAA	Reverse primer for amplification of the *CLV1* sgRNA3 target site
oRT510	TTGCTTTTCCCCTGGACTGG	Forward primer for amplification of the *PDS sgRNA2* target site & DNA sequencing
oLEP127	TGATGATAAGGATGCAGCCTC	Reverse primer for amplification of the *PDS sgRNA2* target site
oDL721	CGGGATTAAGTTTGGTGGAGCAG	Forward primer for amplifying TRV2 for RT-PCR analysis
oDL456	GTCCGTTCCCCTATGGTAAGACAA	Reverse primer for amplifying TRV2 for RT-PCR analysis

Supplementary Table 1. Primers used for sgRNA target site analysis and PCR.
