# Supplementary material for: Virus-mediated, heritable gene editing in groundcherry (Physalis grisea)
Source: Front Plant Sci. 2026 Mar 20;17:1794888. doi: 10.3389/fpls.2026.1794888 (PMC13047112; doi:10.3389/fpls.2026.1794888)
Supplement: Supplementary file 10 [file Table2.pdf]

Vector	sgRNA spacer sequence	sgRNA designation	Target gene
pEE1509	AAGGAGCAGGTAAAGCTTCG	sgRNA1	*PDS*
pEE1511	TTTGGTGGTAGCGAATCCAT	sgRNA2	*PDS*
pRT327	GGTGAAATTCCTCCAAGTCT	sgRNA3	*CLV1*
pRT329	AAATCTTGAAGAACTTAGAT	sgRNA4	*CLV1*
pRT330	AAAGTTCTGGTCTTCTTGAC	sgRNA5	*CLV1*

Supplementary Table 2. Spacer sequences used to target *PDS* and *CLV1*.
