# Supplementary material for: Virus-mediated, heritable gene editing in groundcherry (Physalis grisea)
Source: Front Plant Sci. 2026 Mar 20;17:1794888. doi: 10.3389/fpls.2026.1794888 (PMC13047112; doi:10.3389/fpls.2026.1794888)
Supplement: Supplementary file 11 [file Table3.pdf]

Primer	Sequence (5'-3')	Description
oEE2070	ACACCTGCAACG**AAAC**AAGGAGCAGGTAAAGCTTCGGTTTTAGAGCTAG	Forward primer with *PDS sgRNA1* used to amplify the sgRNA and *FT* mobility sequence from pEE515
oEE2071	ACACCTGCAACG**AAAC**TTTGGTGGTAGCGAATCCATGTTTTAGAGCTAG	Forward primer with *PDS sgRNA*2 used to amplify the sgRNA and *FT* mobility sequence from pEE515
oEE272	TCACCTGCTAGT**CACT**CTAAAGTCTTCTTCCTCCGC	Reverse primer used for single sgRNA vectors
oRT500	CATCGAGCACCTGCTTGG**CACT**TTGGCCATAAGTAACCTTT	Reverse primer used for single sgRNA vectors
oRT501	CACAGGCACCTGCGACC**GCAC**TTGGCCATAAGTAACCTTT	Reverse primer used for multiple sgRNA vectors
oRT479	AAAAGGTCTCG**AAAC**GGTGAAATTCCTCCAAGTCTGTTTTAGAGCTAG	Forward primer with *CLV1* sgRNA1 used to amplify the sgRNA and *FT* mobility sequence from pEE515
oRT503	CACAGGCACCTGCGACC**AAAC**AAATCTTGAAGAACTTAGATGTTTTAGAGCTAGAAATAGC	Forward primer with *CLV1* sgRNA2 used to amplify the sgRNA and *FT* mobility sequence from pEE515
oRT504	CACAGGCACCTGCGACC**AAAC**AAAGTTCTGGTCTTCTTGACGTTTTAGAGCTAGAAATAGC	Forward primer with *CLV1* sgRNA3 used to amplify the sgRNA and *FT* mobility sequence from pEE515

Supplementary Table 3: Primers used to assemble mobile sgRNAs into TRV2 T-DNA. Spacer sequences, gray with wavy underline; AarI or BsaI restriction enzyme sites, black with underline; DNA sequence overhang for Golden Gate cloning into the TRV2 T-DNA backbone pEE083, bold.
